# Maintaining Balance:: Examining Mental Wellness in Black Women during the Menopausal Transition

**DOI:** 10.1093/geroni/igaf122.3809

**Published:** 2025-12-31

**Authors:** Graham McDougall, Elyria Kemp, Xueyan Liu, Candice Sorapuru, Kelly Jones

**Affiliations:** University of Alabama-Tuscaloosa, New Orleans, Louisiana, United States; University of New Orleans, New Orleans, Louisiana, United States; University of New Orleans, New Orleans, Louisiana, United States; Tulane University, New Orleans, Louisiana, United States; Steady Mindz, Kelly Lyn Health, Indianapolis, Indiana, United States

## Abstract

Black women begin the menopausal transition at an earlier age than other women and experience more severe menopausal symptoms such as depression and anxiety. The “Strong Black Woman Schema” (SBWS) is a cultural expectation that these women portray strength and caretaker qualities, while suppressing their emotions. This research had two aims (1) what factors influenced how Black women in midlife perceive, seek, and access mental health care and (2) how these factors could enhance mental well-being. Using a mixed-methods approach, the research integrated qualitative interviews (n = 23) and a segmentation analysis (n = 231) to uncover nuanced insights into the lived experiences of Black women and their mental health decision-making. In the qualitative component the participants ranged from 42–60 in age; the mean age was 49. Approximately 60% of the participants sought mental healthcare. Qualitative findings revealed key themes related to emotional suppression, stigma, cultural expectations, structural barriers, and the role of self-care. In the next phase a survey was distributed online to a pre-established national consumer panel of self-identified Black women using a market research firm in the United States. The quantitative segmentation analysis (N = 231), performed using K-means clustering, identified four distinct groups—Reluctant Avoidants, Evolving Discerners, Practical Engagers, and Steady Seekers—each with unique orientations toward mental health care. Based on these profiles, this research proposes targeted engagement strategies for enhancing access to care. These insights into how Black women navigate mental health care may assist in identifying effective strategies for improving access and support to women.

